# Crebanine, an aporphine alkaloid, induces cancer cell apoptosis through PI3K-Akt pathway in glioblastoma multiforme

**DOI:** 10.3389/fphar.2024.1419044

**Published:** 2024-06-04

**Authors:** Poh-Shiow Yeh, Chien-Te Liu, Chia-Ying Yu, Ya-Chuan Chang, Shu-Yu Lin, Yun-Chen Li, Yu-Ze Luan, Wen-Wei Sung

**Affiliations:** ^1^ Department of Neurology, Chi Mei Medical Center, Tainan, Taiwan; ^2^ School of Medicine, Chung Shan Medical University, Taichung, Taiwan; ^3^ Department of Urology, Chung Shan Medical University Hospital, Taichung, Taiwan; ^4^ Institute of Medicine, Chung Shan Medical University, Taichung, Taiwan

**Keywords:** crebanine, glioblastoma multiforme, brain tumor, natural product, aporphine alkaloid

## Abstract

Glioblastoma multiforme (GBM) is one of the most prevalent and lethal primary central nervous system malignancies. GBM is notorious for its high rates of recurrence and therapy resistance and the PI3K/Akt pathway plays a pivotal role in its malignant behavior. Crebanine (CB), an alkaloid capable of penetrating the blood–brain barrier (BBB), has been shown to have inhibitory effects on proinflammatory molecules and multiple cancer cell lines via pathways such as PI3K/Akt. This study aims to investigate the efficacy and mechanisms of CB treatment on GBM. It is the first study to elucidate the anti-tumor role of CB in GBM, providing new possibilities for GBM therapy. Through a series of experiments, we demonstrate the significant anti-survival, anti-clonogenicity, and proapoptotic effects of CB treatment on GBM cell lines. Next-generation sequencing (NGS) is also conducted and provides a complete list of significant changes in gene expression after treatment, including genes related to apoptosis, the cell cycle, FoxO, and autophagy. The subsequent protein expressions of the upregulation of apoptosis and downregulation of PI3K/Akt are further proved. The clinical applicability of CB to GBM treatment could be high for its BBB-penetrating feature, significant induction of apoptosis, and blockage of the PI3K/Akt pathway. Future research is needed using *in vivo* experiments and other therapeutic pathways shown in NGS for further clinical or *in vivo* studies.

## Introduction

Accounting for 49.1% of primary malignant brain tumors and with a median survival time of less than 2 years, glioblastoma multiforme (GBM) is the most prevalent and lethal primary central nervous system (CNS) malignancy. The incidence of GBM is more common in males and increases after the age of 40 (Stupp et al., 2005; Stupp et al., 2017; Ostrom et al., 2021; Salari et al., 2023; Schaff and Mellinghoff, 2023). GBM originates primarily in astrocytic glial cells and has been shown to have a high amount of uncontrolled cellular proliferation, angiogenesis, and invasion and intense resistance to cell death, apoptosis, and necrosis (Furnari et al., 2007). Despite rapid advances in standard therapies, including surgical resection, chemotherapy with temozolomide (TMZ), and radiotherapy, GBM remains highly refractory to therapy, and the recurrence of tumors is inevitable (Schaff and Mellinghoff, 2023). Notably, unlike other intracranial tumors, GBM is largely unresponsive to molecularly targeted therapies. Due to its intense resistance to death-inducing stimuli, understanding its specific molecular mechanisms is crucial for identifying novel therapeutic approaches to GBM (Kornienko et al., 2013; Mellinghoff and Cloughesy, 2022).

It has been postulated that the characteristic of GBM’s intense resistance to cell death is associated with genetic alterations in regulatory molecules involved in mitogenic signaling, especially those involving the signaling pathway of phosphoinositide 3-kinase (PI3K)/phosphatase and tensin homolog (PTEN)/protein kinase B (Akt) induced by hepatocyte growth factor/hepatocyte growth factor receptor (MET), the death receptor systems of the TNFR1 and TNF-related apoptosis-inducing ligand, and the anti-apoptotic BCL-2 family. It has been suggested that these mechanisms play a pivotal role (Chakraborty et al., 2013; Cruickshanks et al., 2017; Deng et al., 2021; Yu et al., 2022). The PI3K/Akt signaling pathway has been considered essential for GBM proliferation, migration, metabolism, angiogenesis, survival, and cancer pathogenesis. Previous studies have shown that PTEN, a tumor suppressor antagonizing PI3K/Akt signal transduction and inactivating 50% of GBMs, leads to uncontrolled PI3K/Akt signaling in GBM (Knobbe and Reifenberger, 2003; Jhanwar-Uniyal et al., 2022).

Crebanine (CB), an aporphine alkaloid, is a natural product found in *Stephania cephalantha, Stephania hainanensis*, and *Stephania abyssinica*. CB has demonstrated inhibitory effects not only on immune regulation but also on multiple cancer cell lines. In previous studies on macrophages, CB suppressed the PI3K/Akt pathway and MAPK pathway of lipopolysaccharide (LPS)-induced toll-like receptor downstream reactions, thus inhibiting the activation of nuclear factor kappa B (NF-κB) and activator protein-1 (AP-1), resulting in a decrease in proinflammatory factors interleukin-6, tumor necrosis factor α (TNF-α), inducible nitric oxide, and prostaglandin E2 (Intayoung et al., 2016). It also exhibited antitumor activities via the inhibition of the NF-κB signaling pathway and its downstream gene expression, resulting in a reduction in TNF-α-induced cell proliferation, invasion, and death (Yodkeeree et al., 2014; Intayoung et al., 2016; Mon et al., 2018; Wang M. et al., 2022). Moreover, previous research on the ability of substances to penetrate the blood–brain barrier (BBB) (Shaker et al., 2020) indicates that CB is capable of penetrating the BBB, making it a potential candidate for GBM treatment.

Given its potential as a BBB-penetrating PI3K inhibitor and anti-cancer agent, CB may open promising future avenues for these potentially critical signaling pathways in therapeutic intervention. Our aim was to investigate the efficacy and mechanisms of CB treatment on GBM. This study is the first to elucidate the anti-tumor role of CB in GBM, providing new perspectives and exploring the potential value of alternative adjuvant therapies for GBM.

## Materials and methods

### Cell culture

Human GBM cell lines LN229 (Elabscience Biotechnology Inc., United States), T98G (Riken Cell Bank, Japan), and U87MG (Bioresource Collection and Research Center, Taiwan) were cultured and stored according to the suppliers’ instructions. The three cell lines were maintained in a high-glucose (4.5 g/L) DMEM medium, RPMI-1640 medium, and MEM medium, respectively. All media were supplemented with 10% fetal bovine serum, 100 U/mL penicillin, 100 μg/mL streptomycin, 2 g/mL NaHCO_3_, 1 mM sodium pyruvate, and 0.1 mM NEAA. All reagents were obtained from Gibco Life Technologies Ltd. (Paisley, United Kingdom). The cell lines were cultivated in a 37°C incubator at 5% CO_2_ (Wang et al., 2021; Wang S. C. et al., 2022).

### MTT assay

An MTT assay was used to detect cytotoxicity and cell growth. Briefly, 10^4^ cells were seeded onto 96-well plates and exposed to different concentrations of CB (MedChemExpress, United States) for 48 h. An MTT reagent (0.5 mg/mL) was added to each well and incubated for 3 h at 37°C. The reaction was stopped by removing the supernatant, followed by dissolving the formazan product in DMSO. Optical density was measured with an ELISA reader using a 570 nm filter. Each experiment was performed in triplicate, and the cell viability of each group was calculated and analyzed in comparison to a control (Wang et al., 2021; Wang S. C. et al., 2022).

### Colony formation

In each well of a six-well plate, 3,000 cells were incubated overnight to allow for cell attachment. The following day, the cells were exposed to CB (0, 50, 100, and 200 μM) for 24 h. After 7 days, colonies were fixed with 95% ethanol and stained with crystal violet (1 mg/mL). Each experiment was performed in triplicate, and the colony counts of each group were calculated (Wang et al., 2021; Wang S. C. et al., 2022).

### Flow cytometry analysis

Cell cycle phase distribution and apoptosis percentage were accessed using a flow cytometer (FACSCanto™ II Cell Analyzer; BD Biosciences, United States). Cells were treated with CB (0, 50, 100, and 200 μM) for 48 h. To analyze the cell cycle phase distribution, the cells were fixed in 70% (v/v) cold ethanol overnight. After being washed with PBS, cells were incubated in PBS containing 0.4 μg/mL PI and 0.5 mg/mL RNase for 30 min at 37°C in the dark. To quantify apoptotic rates, an Annexin V/PI apoptosis detection kit (Elabscience Biotechnology Inc., United States) was used according to the manufacturer’s protocol. Cells were stained with FITC-Annexin V and PI in the dark for 15 min at room temperature. All samples were performed in triplicate. Individual cell suspensions were analyzed using flow cytometry, and cell profiles were analyzed using FlowJo software (BD Biosciences, United States) (Wang et al., 2021; Wang S. C. et al., 2022).

### Hoechst 33342 staining

Cell apoptosis was observed using Hoechst 33342 staining. Cells were seeded in a six-well plate and treated with CB (0, 50, 100, and 200 μM) for 24 h. Next, the cells were incubated with Hoechst 33342 (10 ug/mL, Invitrogen, United States) for 20 min. Cell morphological changes relating to apoptosis were observed, and images were captured using fluorescence microscopy (ImageXpress PICO, Molecular Devices, United States), with an excitation wavelength of 350–390 nm and an emission wavelength of 420–480 nm. Apoptotic cells emitted blue fluorescence and exhibited morphological changes in the nuclei typical of apoptosis. For quantification of apoptotic cells, five random visual fields were randomly selected to calculate the percentage of apoptosis (Wang et al., 2021; Wang S. C. et al., 2022).

### Next-generation sequencing (NGS)

The GBM cell lines LN229, T98G, and U87MG were treated with CB (0 and 200 μM) for 24 h, and the total RNA was extracted using Trizol^®^ Reagent (Invitrogen, United States) in accordance with the manual for instruction. Genomics (Taiwan) performed the sample preparation, library preparation, sequencing, alignment, and differential expression analysis, and the analyses were conducted according to the official protocol. Genes with a *p*-value ≤0.05 and ≥ 2-fold changes were considered to have a significantly differential expression. The analysis of the overrepresented cell cycle–related gene ontology (GO) and enrichment KEGG pathway used the compareCluster function in the R package clusterProfiler. The top 10 overexpressed GO terms and KEGG pathways were visualized, and the cut-off criterion was a *p*-value <0.05.

## Results

### CB induced cell death and decreased the viability and colonogenicity of GBM cells

**FIGURE 1:**
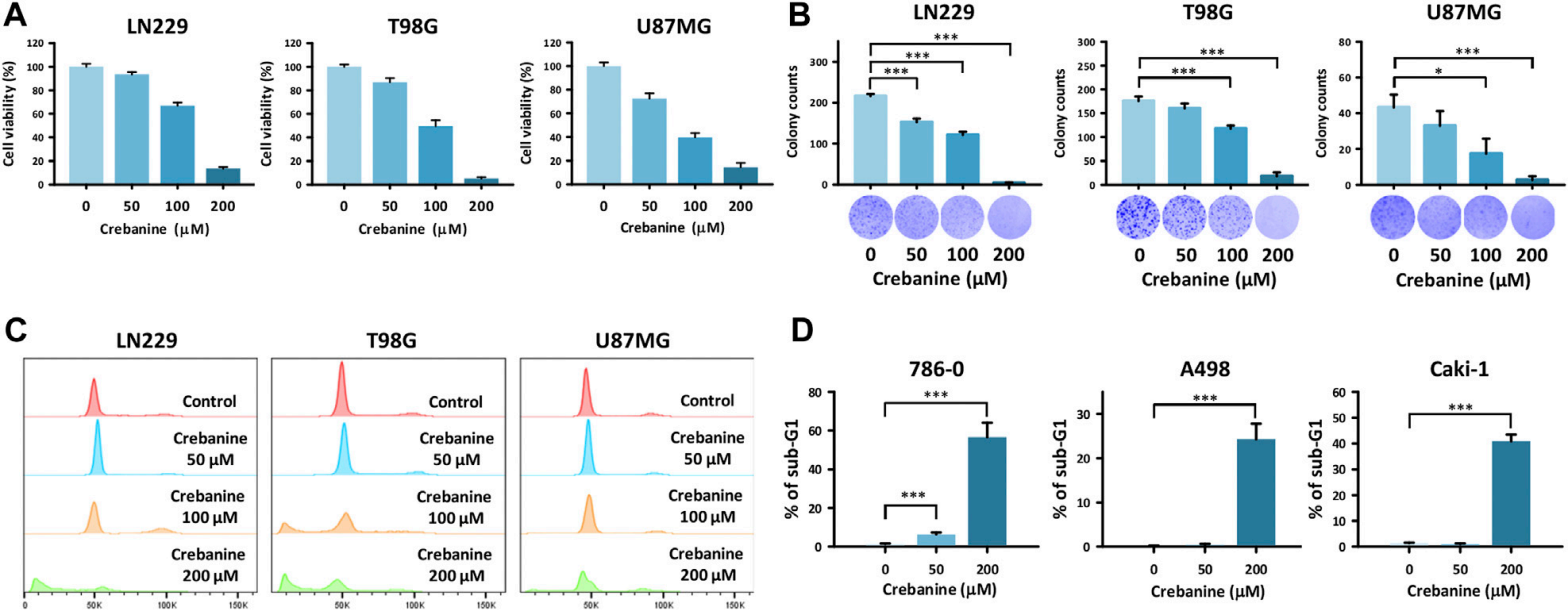
CB treatment–induced cell death in GBM cells. **(A)** Reduced viability of GBM cells was shown after CB treatment in the MTT assay. **(B)** The reduction in colony formation of GBM cells was shown after CB treatment in the colony formation assay. **(C,D)** Increased proportions of the sub-G1 group in GBM cells were shown after CB treatment in the cell cycle analysis. Data are shown as mean ± S.D. (*p < 0.05; **p < 0.01; ***p < 0.001).

The results of the MTT and clonogenic assays indicate that the viability and colony formation of CB cells decreased dose-dependently in all cell lines (Figures 1A, B). Cell cycle analysis revealed an increase in the sub-G1 group in all GBM cell lines (Figures 1C, D). These results show that CB inhibited cell growth and caused cell death in GBM cells. In addition, apoptosis played a role in these phenomena.

### Annexin V/PI double staining and Hoechst 33342 staining revealed the proapoptotic property of CB treatment in GBM cells

**FIGURE 2:**
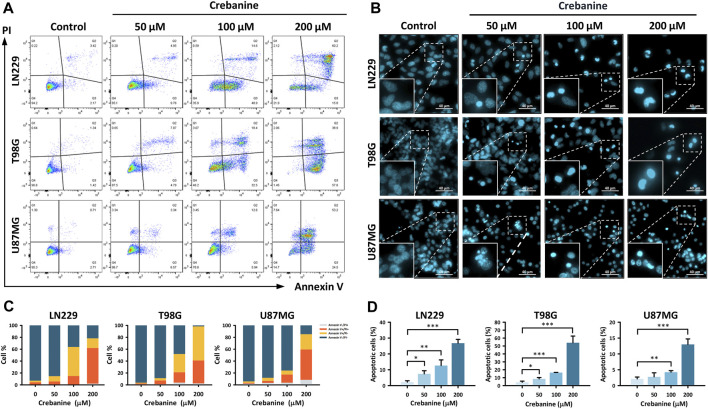
CB caused apoptosis in GBM cells. **(A,C)** Annexin V/PI double staining demonstrated the proportions of early apoptosis, late apoptosis, and necrosis in CB-treated GBM cells. The results are also shown in bar charts. **(B,D)** Hoechst 33342 staining demonstrated morphological changes in apoptotic GBM cells after CB treatment. The results, as represented in the bar charts, show increased proportions of apoptotic GBM cells after CB treatment. Data are shown as mean ± S.D. (*p < 0.05; **p < 0.01; ***p < 0.001).

To ensure that apoptosis was triggered in CB-treated GBM cells, Annexin V/PI double staining and Hoechst 33342 staining were carried out (Figure 2). In Annexin V/PI double staining, the proportion of late apoptotic (Annexin V+/PI+) cells increased dose-dependently after CB treatment (Figures 2A, C). For the Hoechst 33342 staining, the distributions of positive staining GBM cells increased dose-dependently after CB treatment in comparison with the control groups (Figures 2B, D). Therefore, we suggest that apoptosis is the major type of cell death in GBM cells induced by CB treatment.

### Changes in the expression of genes related to nuclear division, mitosis, and the cell cycle are shown in CB-treated GBM cells

**FIGURE 3:**
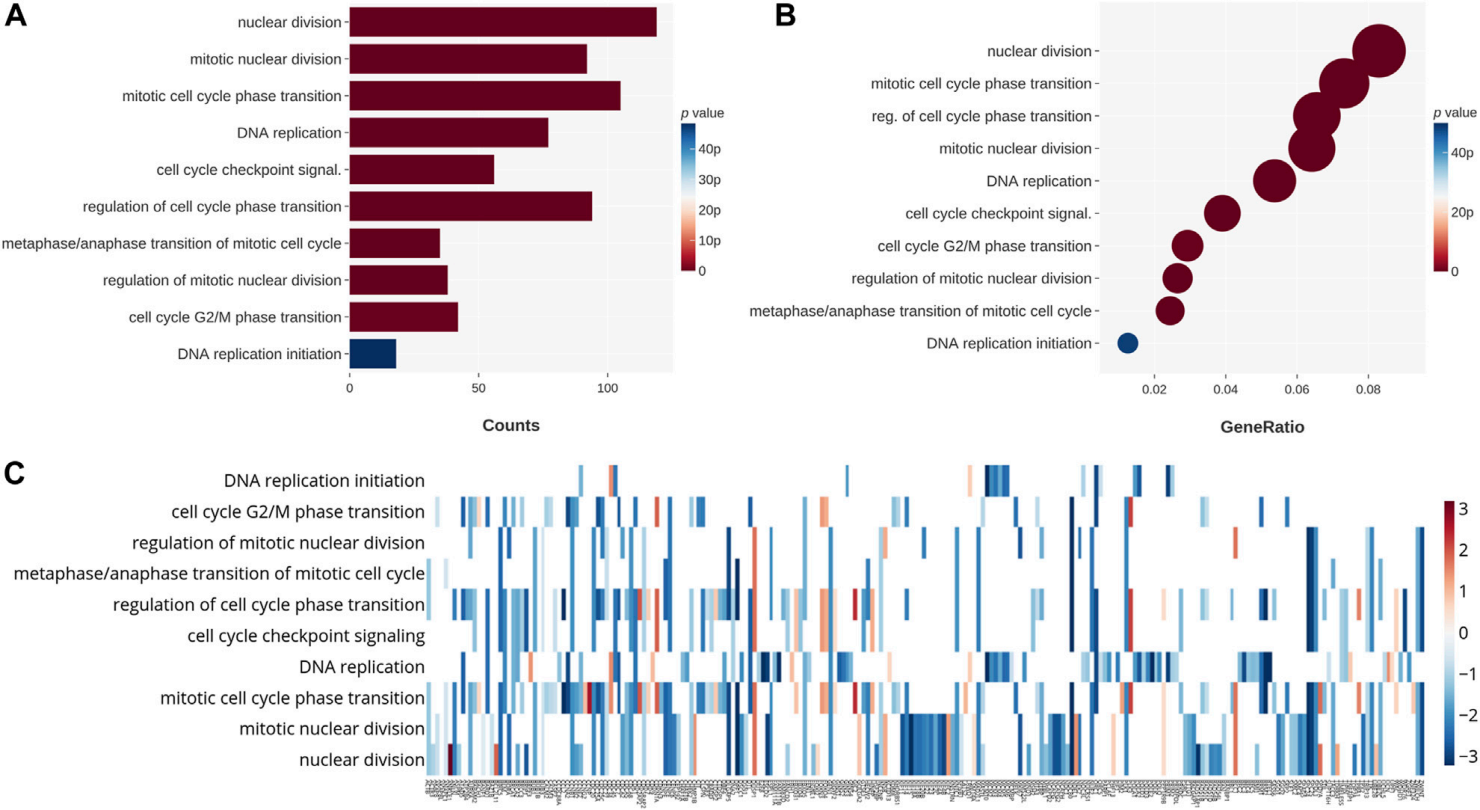
The 24-h CB-treated GBM cells were tested by NGS with the GO database. These genes are categorized on the basis of their functions. **(A)** The categories that changed significantly in count are shown on the chart and sequenced in the order of their *p* values. **(B)** The categories that changed significantly in ratio are shown on the chart and sequenced in the order of their ratios. Their *p* values are shown in color. **(C)** The heat map shows in color the *p* values for changes in gene expression. The categories for these genes are also shown.

To further understand the mechanism behind the downregulation of viability and colonogenicity, we not only investigated the apoptosis pathway but also tried to research other potential mechanisms with NGS. Cell cycle–related genes of the GO database were examined in relation to the 24-h CB-treated GBM cells. It was clear that enormous changes occurred in nuclear division, mitosis, cell cycle transition, and DNA replication. In Figure 3A, genes related to nuclear division changed most significantly, followed by mitosis, DNA replication, and cell cycle transition. As for the ratio (Figure 3B), the order of categories was highly similar to that sequenced by the *p* values in Figure 3A, except for the genes related to the regulation of cell cycle phase transition, which ranks as high as the categories of genes related to mitosis and higher than DNA replication. In Figure 3C, the genes for each category are shown, and it is obvious that most genes were downregulated. Therefore, we suggest that CB treatment of GBM cells not only causes apoptosis but also leads to a decline in nuclear division, mitosis, and DNA replication and an increase in cell cycle arrest. These effects may have collaboratively caused the decrease in the viability and colonogenicity of CB-treated GBM cells.

### Apoptosis could account for the death and decreased viability and colonogenicity of CB-treated GBM cells. Cell cycle arrest and autophagy were also observed using NGS

**FIGURE 4:**
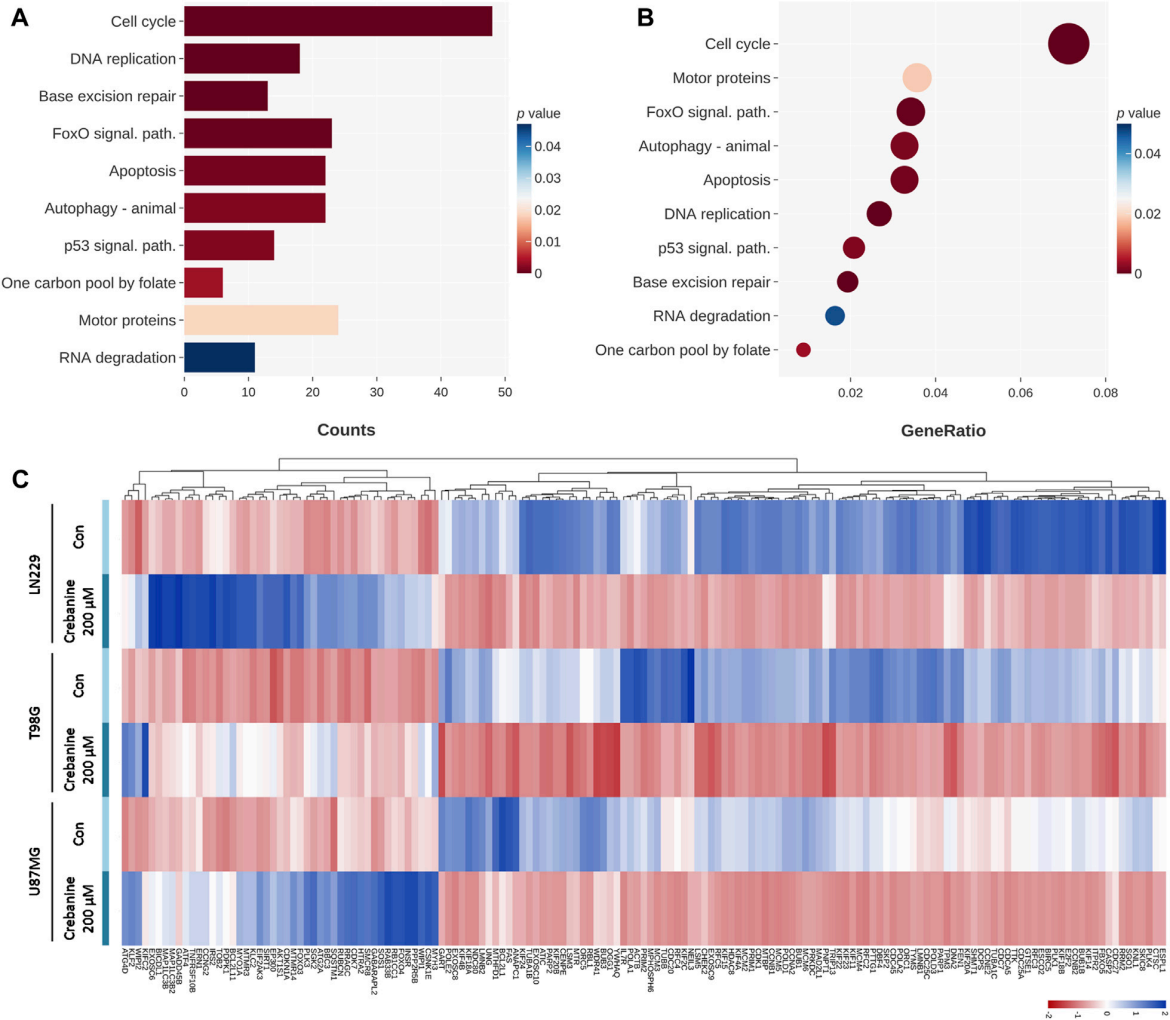
The 24-h CB-treated GBM cells were tested by NGS with the KEGG database. These genes are categorized on the basis of their functions. **(A)** The categories that changed significantly in count are shown on the chart and sequenced in order of their *p* values. **(B)** The categories that changed significantly in ratio are shown on the chart and sequenced in the order of ratio. Their *p* values are shown in color. **(C)** The heat map shows in color the *p* values of the changes in gene expression.

The genes in the KEGG database were also used to ensure the dependability of our findings. From the charts, the changes in the expression of genes of the cell cycle and DNA replication were large, which also happened with the charts of the GO database. However, there were still differences between the results for the KEGG database and the GO database. First, the changes in the genes for autophagy, apoptosis, and the p53 signaling pathway were significant in the KEGG database, whereas the GO database did not show these categories. Second, genes specifically related to mitosis were not shown in the charts for the KEGG database but are one of the groups that changed most significantly in the GO database. Third, the genes concerning the fundamental metabolisms of cells showed a significant change in the KEGG database, including those for the FoxO signaling pathway, one carbon folate cycle, motor proteins, and base excision repair. These genes could be related to the apoptosis, cell cycle arrest, and autophagy of GBM cells. In Figure 4A, the changes in the genes related to the cell cycle were the most significant, followed by DNA replication, base excision repair, the FoxO pathway, apoptosis and autophagy, the p53 pathway, and the folate cycle. In terms of the ratio (Figure 4B), cell cycle–related genes still topped the chart, and the order of the categories shared a high similarity with Figure 4A. The most notable difference was that motor protein genes ranked second in change ratio, while they ranked second last in *p*-value. Simultaneously, the genes regarding base excision repair ranked third for *p*-value but third last for change ratio. This may mean that the motor proteins and base excision repair were both impacted by CB treatment and were factors in downregulation but not major inducers. Figure 4C shows the change in gene expression of the categories in Figures 4A, B. It is obvious that the gene expression of the GBM cells changed substantially after CB treatment. In summary, we suggest that cell cycle arrest and autophagy may contribute to cell death and reduced viability and colonogenicity. The apoptosis of these cells, previously proved by Annexin V/PI double staining and Hoechst 33342 staining, was also observed via NGS.

### The expression of proapoptotic proteins is demonstrated by western blotting of 24-h CB-treated GBM cells

**FIGURE 5:**
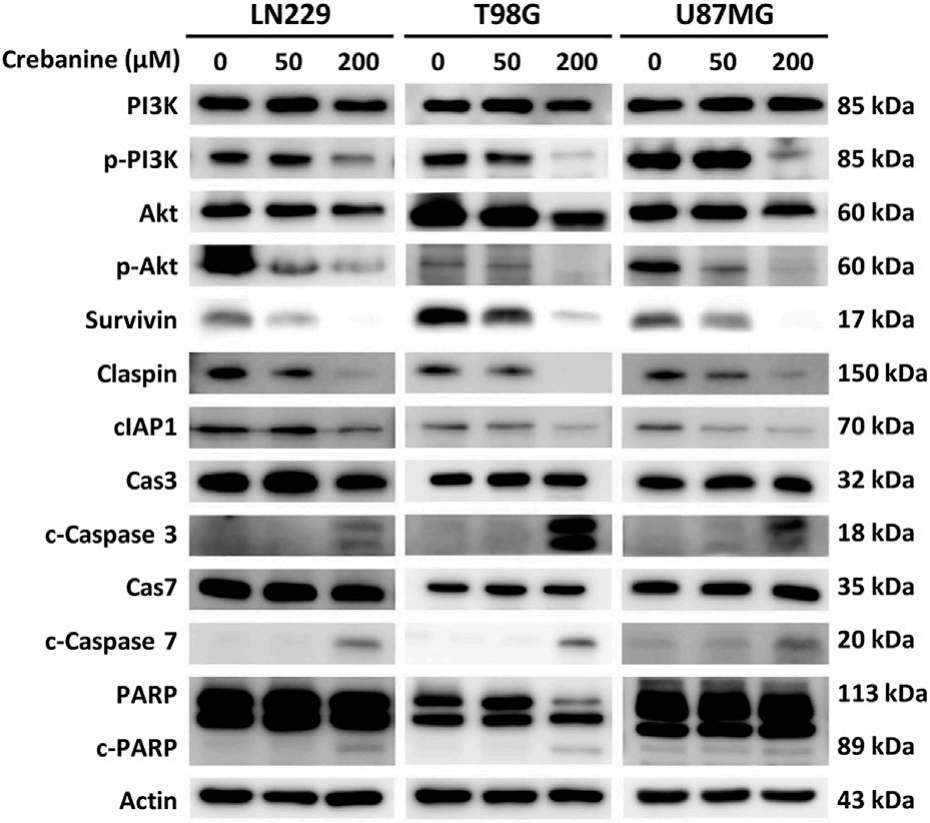
Western blotting revealed the expression of apoptotic proteins of the PI3K/Akt signaling pathway and caspase cascade in CB-treated GBM cells.

To ensure the appearance of apoptosis in CB-treated GBM cells at the protein level, CB-treated GBM cells were examined using western blotting. Proteins of the PI3K/Akt pathway and the execution phase of apoptosis were examined (Figure 5). We observed the downregulation of antiapoptotic proteins, including phosphorylated PI3K, phosphorylated Akt, survivin, claspin, and cellular inhibitor of apoptosis protein 1 (cIAP1), as well as the upregulation of proapoptotic proteins, including cleaved caspase 3, cleaved caspase 7, and cleaved poly (ADP-ribose) polymerase (PARP). These results provide evidence for CB’s property of apoptotic induction in GBM, which could be beneficial for the treatment of GBM.

## Discussion

In the present study, we investigated the influence of CB treatment on GBM cell lines. It is obvious that CB decreases the viability and clonogenicity of GBM, and apoptosis plays an important role in this. In addition, through NGS and western blotting, the proapoptotic ability of CB treatment was shown at the gene and protein levels, which could be associated with PI3K/Akt pathway. This is the first study to reveal the anti-cancer effect of CB, a postulated BBB-penetrating immunoregulator and anti-cancer agent, on the most prevalent and lethal primary CNS cancer, GBM, and to examine the mechanisms involved.

To begin with, we examined the impact of CB treatment on GBM treatment at the cellular level through a series of experiments. The findings from the MTT and clonogenic assays showed CB’s ability to curb the viability and clonogenicity of GBM cells. Through cell cycle analysis, Annexin V/PI double staining, and Hoechst 33342 staining, it was shown that CB treatment successfully induces apoptosis in GBM cells, which could be the reason for its inhibition of viability and clonogenicity. These results are similar to previous studies of CB on other cancer cells. In 2010, a study on CB revealed that it inhibits viability and induces cell cycle arrest in the erythroleukemia cell K562 and the small cell lung cancer cell GLC4. The anti-viability effect of CB is the best of all extracts from *Stephania venosa* in terms of half maximal inhibitory concentration (IC_50_) (Nantapap et al., 2010). In 2012, a study on the effect of CB on multiple cancer cell lines showed promising results. CB significantly inhibited the viability of human leukemic cells HL-60, U937, and K562, the lung cancer cell HT1080, and cervix cancer cell lines KB-31 and KB-V1 in a dose-dependent manner. In HL-60 and U937 cells, apoptosis and cell cycle arrest pathways were induced after CB treatment. Furthermore, for HL-60, the cell line most sensitive to CB among all those tested in the study, the pathways were identified with western blotting (Wongsirisin et al., 2012). Our western blotting of the apoptosis pathway revealed that the same mechanism was also expressed in GBM cells. In 2014, a study showed that the dose-dependent synergic effect of TNF-α and CB significantly induced apoptosis in the human lung adenocarcinoma cell A549 (Yodkeeree et al., 2014). In 2020, a study on human gastric cancer SGC-7901 cells proved that apoptosis, cell cycle arrest, and autophagy are induced by CB treatment, which correlates highly with our findings for NGS (Wang et al., 2020). The present study shows the therapeutic potential of CB for its induction of GBM apoptosis.

To comprehend CB’s effect on GBM cells, NGS was conducted to examine changes in RNA expression. The results from the KEGG database and the cell cycle–related genes of the GO database showed that the most significantly changed genes in expression were those related to the cell cycle, including mitosis and DNA replication, apoptosis, the FoxO pathway, and autophagy.

The expressional change of cell cycle–related genes was drastic, including all the 10 top significant results from cell cycle–related genes of the GO database and the first and second most significant items from the KEGG database in terms of gene count, namely, “cell cycle” and “DNA replication.” As mentioned above, the effect of CB on cell cycle arrest has also been demonstrated in previous studies. G0/G1 cell cycle arrest was induced in human leukemia, erythroleukemia, and small cell lung cancer cells, and G2/M cell cycle arrest was induced in human gastric cells. Although our cell cycle analysis revealed that this effect is not present in GBM at the cellular level, NGS showed a significant change in gene expression in relation to the cell cycle. Future studies on the reason for its lack of significance at the cellular level may be needed.

In addition, it is obvious that the FoxO signaling pathway and p53-related genes are upregulated by CB, and they could be associated with the apoptosis pathway. It has been shown in a previous study that apoptosis is one of the downstream pathways of FoxO signaling, which could cause apoptosis both directly and indirectly via crosstalk with p53 (Zhang et al., 2011). As mentioned above, a few previous studies have also shown the proapoptotic effect of CB in human leukemia, lung adenocarcinoma, and gastric cancer cells. Our study not only shows that apoptosis, the most potential therapeutic pathway of CB, is also induced in GBM but also finds another possibly related mechanism, the pathways of FoxO and p53, behind CB’s proapoptotic property. In addition, upregulated autophagy gene expression may also be a consequence of the FoxO pathway, since it has been proven that the FoxO pathway leads to autophagy (Cheng, 2019). Although autophagy plays roles in both tumor promotion and suppression, our results in cell viability and clonogenicity show a tendency for cell death and inhibition of proliferation (Cheng, 2019; Yang et al., 2023). A similar result was found in human gastric cells, as mentioned previously.

In addition, NGS showed a significant decrease in the gene expression of base excision repair and motor proteins. This decrease may undermine GBM cells’ ability to function normally, collaboratively reducing the viability and proliferation of GBM (Poletto et al., 2017), especially given that kinesin family members, the motor proteins downregulated in the result of NGS, have been found to be connected to and even advantageous for the proliferation of GBM cells (Wang L. B. et al., 2022; Wang L. et al., 2022). NGS has revealed several anti-cancer mechanisms for the CB treatment of GBM cells. We also explored the influence of CB in apoptosis on the protein level through western blotting to obtain a deeper understanding; this is one of the most clinically applicable mechanisms demonstrated in NGS findings and showed significant results in our experiments at the cellular level. Future research on other clinically applicable pathways and the lack of significance of cell cycle arrest in experiments at the cellular level may be needed.

Western blotting showed the downregulation of phosphorylated PI3K and Akt and the upregulation of the execution phase of apoptosis, the caspase cascade. The upregulation of caspases could be explained as the downstream reaction of inhibition of the PI3K/Akt pathway. The ability of CB to inhibit PI3K was previously reported in research on macrophages, in which CB suppressed the LPS-induced production of proinflammatory factors via the PI3K/Akt/NF-κB or AP-1 pathway (Intayoung et al., 2016). In addition to producing proinflammatory factors, the PI3K/Akt pathway has been shown to suppress apoptosis via inactivation of caspase 3/7 and activation of B-cell lymphoma 2 (Bcl-2) protein in head and neck squamous cell carcinomas (Su et al., 2022). Another study on the influence of CB on the hepatocellular carcinoma cell HepG2 showed inhibition of the PI3K/Akt/FoxO3a pathway, possibly inducing the mitochondrial apoptosis pathway (Tan et al., 2023). Our study further demonstrated that CB inhibits the PI3K/Akt pathway and could thus induce apoptosis in GBM.

In GBM cells, the PI3K pathway plays a pivotal role in carcinogenesis, survival, proliferation, etc. (Knobbe and Reifenberger, 2003; Jhanwar-Uniyal et al., 2022). Consequently, attempts have been made to treat GBM through this pathway, although its clinical applicability is still limited or unexplored. SINI-WCJ-33, an alkaloid, has been shown to curb the proliferation, colony formation, migration, and invasion of GBM cells. It has been found to activate autophagy and mitophagy via the PI3K pathway. Future research on its clinical applicability is still needed (Li and Xu, 2023). Buparlisib, a BBB-penetrating panPI3K inhibitor, has shown potent inhibitory effects on GBM, both *in vitro* and *in vivo*. However, it only showed limited efficacy in phase II studies of recurrent GBM (Wen et al., 2019; Barzegar Behrooz et al., 2022). Some research on GBM aims at repurposing drugs originally intended for other diseases. Metformin and chlorpromazine both target the PI3K pathway’s downstream AMP-activated protein kinase/mammalian target of the rapamycin (mTOR) pathway, which is responsible for most of the procancer behavior of the PI3K pathway. However, GBM can counteract their effect through glutaminase (Barzegar Behrooz et al., 2022). Therefore, experiments on the combination of mTOR and GLS inhibitors have been conducted, and they have shown anti-tumor effects in xenograft models (Tanaka et al., 2015; Barzegar Behrooz et al., 2022). Several studies on the PI3K pathway in GBM cells have been conducted. Some have shown remarkable efficacy in preclinical settings, but their clinical applicability still requires examination. The present study introduces a novel anti-GBM drug, CB, which not only blocks the PI3K pathway but has also been shown to have inhibitory effects on the NF-κB signaling pathway in previous studies. We successfully demonstrated its significant proapoptotic effect and identified a possible underlying mechanism, including the PI3K, FoxO, and p53 pathways. Further research is needed to explore its clinical applicability and effects on other therapeutic pathways shown by NGS.

There are some limitations to our study. First, experiments using only a few cell lines may fail to represent all types of GBM cells. Second, an *in vivo* study is needed to compensate for the natural deficiencies of *in vitro* studies. The influence of the tumor microenvironment and its adverse effects remain unknown. The actuality of CB’s ability to penetrate the BBB also needs to be shown through *in vivo* experiments. Third, the dosages used in our research, though referenced from published studies, are relatively high (Cui et al., 2022; Tan et al., 2023). The toxicity of high-dose CB on normal healthy cells is a significant concern. Despite the higher half-maximal inhibitory concentration found in normal embryonic lung cells compared to tumor cells, further detailed research is necessary (Makarasen et al., 2011). Further research on other GBM cell lines and animals is thus needed to ascertain the effect of CB treatment. We hope this novel drug will be beneficial to numerous patients suffering from this deadly disease.

## Data Availability

The original contributions presented in the study are included in the article. The raw data can be found here: https://figshare.com/projects/GBM_CB/207316.
